# Evaluation of Protein Concentration and Limiting Amino Acids Including Lysine and Met + Cys in Prestarter Diet on Performance of Broilers

**DOI:** 10.1155/2012/394189

**Published:** 2012-09-29

**Authors:** Mohsen Farkhoy, Mehrdad Modirsanei, Omid Ghavidel, Majid Sadegh, Sadegh Jafarnejad

**Affiliations:** ^1^Department of Animal and Poultry, Health and Nutrition, Faculty of Veterinary Medicine, University of Tehran, P.O. Box 14155-6453, Tehran, Iran; ^2^Faculty of Veterinary Medicine, University of Tehran, P.O. Box 14155-6453, Tehran, Iran

## Abstract

Four experiments were conducted, in two stages, to evaluate protein and limiting amino acids' (lysine and methionine + cystine) levels in pre-starter diets on broilers' performance. In each experiment of Stage 1, 640 new-born male Ross 308 cockerels were randomly allocated to eight dietary treatments with a 2 × 4 factorial arrangement. In experiment 1-1, two levels of crude protein (CP: 21% and 23.2%) and four levels of Lys (1.2, 1.3, 1.4, and 1.5%) and in experiment 1-2, two levels of CP (21 and 23.2%) and four levels of Met + Cys (0.85, 0.90, 0.95, and 1.00%) were used. In Stage 2, the optimum levels of Lys and Met + Cys obtained from Stage 1 (1.3 and 1.5% Lys, 0.90 and 1.00% Met + Cys in experiment 1-1 and 1-2, resp.) with two levels of CP (21 and 23.2%) were used in two separate simultaneous experiments with a 2 × 2 factorial arrangement for male and female birds. The levels of CP significantly influenced BWG and FCR in experiment 1-1. Dietary levels of Lys affect BWG (experiment 1-1) and FI (experiments 1-1 and 2-1) significantly. In experiments 1-2 and 2-2, the different levels of Met + Cys did not affect BWG, FI, and FCR of male or female broilers. The results of these experiments indicated that the optimal level of dietary protein and Lys were 23.2% and 1.5%, respectively. Diets with 1% Met + Cys caused optimal performance.

## 1. Introduction

Worldwide poultry production has increased significantly over the past fifty years to accommodate rising demand. Broiler chicks grow rapidly and typically receive diets high in protein or amino acids [1]. It is common practice in the poultry industry to provide varying diets during the growing period. 

Protein is an essential constituent of all tissues of animal body and has major effect on growth performance of the bird [2]. A better understanding of the nutritional requirements of amino acids allows a more precise nutrition, offering the possibility for the formulator to optimize the requirement of at least minimum levels of crude protein by essential amino acids requirements, generating better result and lower costs for the producer [3].

Feeding high amino acid density diets improves feed conversion and increases weight gain and breast meat yield of broiler chickens [4].

Methionine + cystine (total sulfur amino acid = TSSA) perform a number of functions in enzyme reactions and protein synthesis. Methionine is an essential amino acid for poultry and has an important role as a precursor of cystine [5].

Methionine is usually the first limiting amino acid in most of the practical diets for broiler chicken [6, 7]. 

Lysine is often one of the limiting amino acids in broiler diets. As such, it used as the reference amino acid to which all other essential amino acids are rationed in the ideal amino acid pattern [8]. Therefore, it is crucial to obtain an accurate Lys and Met + Cys requirement to support optimum growth of fast-growing commercial broilers. 

Many poultry nutritionists use the levels recommended by the National Research Council [9] as a guideline in establishing their own amino acid requirements.

Although most amino acid requirements established by the NRC [9] considered safe estimates for broiler chicks, some researchers have indicated that the NRC [9] lysine requirement for chicks is too low [10–14]. Other studies have reported that Met + Cys levels should be above NRC [9] recommendations [15–17]. The present study aims to determine the protein, lysine, and methionine + cystine requirements for growth and feed efficiency for broiler chickens 1–10 days of age.

## 2. Materials and Methods

Trials were conducted in Amin-Abad Research Institute, Faculty of Veterinary Medicine at the University of Tehran,Tehran, Iran.This study was conducted in two stages and two experiments each stage.

*Stage 1*. Six hundred and forty one-day-old male broiler chicks of a commercial strain (Ross 308) randomly allocated to eight treatments with 80 chicks per treatment. Each treatment consisted of four replicates of 20 birds each. Birds were kept in battery cages. Stage 1 was carried out in two separate experiments. In experiment 1-1, a 2 × 4 factorial arrangement was used with two levels of crude protein (CP: 21% and 23.2%) and four lysine levels (Lys: 1.2, 1.3, 1.4, and 1.5%) (Table 1).

In experiment 1-2, there were two levels of CP (21 and 23.2%) and four levels of methionine + cystine (Met + Cys: 0.85, 0.90, 0.95, and 1.00%) according to a 2 × 4 factorial arrangement (Table 2).

In both experiments, chicks were reared in conventional broiler chicks' management in an environmentally controlled house. They were reared up to 10 days of age. Birds had *ad libitum* access to feed and water and lighting was provided continuously. Room temperature adjusted at 32°C in the first week of age and decreased to 28°C in the second week. Relative humidity also adjusted in 60%. All the chicks were immunized against infectious bronchitis at 1 d of age via coarse spray and Newcastle disease at 7 d of age via drinking water. The average initial body weight was 50 g. All birds received a common prestarter diet in mash form up to 10 days of age.

*Stage 2*. In stage 2, the levels of Lys and Met + Cys which had better performance in stage 1 (1.3 and 1.5% Lys in experiment 1-1 and 0.90 and 1.00% Met + Cys in experiment 1-2) with two levels of CP (21 and 23.2%) were used for both male and female broilers in two separate simultaneous experiments (experiment 2-1 and experiment 2-2), with a 2 × 2 factorial arrangement. In this stage, 3200 one-day-old Ross 308 male and female broiler chicks (1600 males and 1600 females) raised in floor pens. The chicks divided into 8 treatments, with 4 replicates per each treatment of 50 birds. All conditions in this stage were similar to stage 1. Body weights gain (BWG) and feed intake (FI) were recorded at the end of 7 and 10 days of age, and feed conversion ratio (FCR) was calculated. 

All of the data were subjected to analysis of variances using the general linear model (GLM). The means of variables were compared according to Duncan's multiple range test, when the significant differences were observed [18]. The level of significance was established at *P* < 0.05. 

## 3. Results

*Stage 1*. In experiment 1-1, the effects of dietary CP and Lys levels on BWG, FI, and FCR in 1–7 and 1–10 d of age are shown in Table 3. Dietary levels of CP significantly influenced BWG and FCR (BWG: 1–7 d and 1–10 d; FCR: 1–10 d) (*P* < 0.05). The BWG was significantly higher in broilers fed with 23.2% CP than diets with 21% CP at all ages examined. During 1–7 d and 1–10d, maximum BWG was observed in birds fed on diet containing 23.2% CP, T1 (Table 3). There was no significant difference for FI between treatments with various levels of CP (*P* > 0.05). The treatments with higher level of CP showed better FCR in 1–10 d (*P* < 0.05) but no difference in 1–7 d (*P* > 0.05). In this study, dietary levels of Lys increased BWG on 1–7 and 1–10 d, significantly (*P* > 0.05). A significant difference was observed in FI on 1–7d; it means that higher Lys levels resulted in higher FI. During 1–7 d, the highest and lowest FI were belonged to the chicks received 1.5% and 1.2% Lys, respectively, (*P* < 0.05). There was no significant difference for FI on 1–10 d (*P* > 0.05).

The effect of Lys levels on FCR was not significant neither on 1–7 d nor on 1–10 d of age (*P* > 0.05). Although no significant differences were observed among FCR of dietary treatments throughout the experimental period (*P* > 0.05), the birds fed diet including 23.2% CP and 1.5% Lys had the best FCR on 1–7 d and 1–10 d of age (Table 3). 

The effects of the levels of dietary CP or Met + Cys on broilers' performance, in exp. 1-2, are shown in Table 4. The levels influence of CP or Met + Cys on BWG, FI, and FCR were not significant in 1–7 d and 10 d (*P* > 0.05). There was significant differences among treatments in BWG and FI (1–10 d), and FCR (1–7 and 1–10 d). The highest BWG and the lowest FCR in 1–7 d and 1–10 d were observed in the birds fed diet including 23.2% CP and 1.0% Met + Cys.

*Stage 2*

*Experiment  2-1*. The CP had no significant effect on BWG, FI, and FCR at 10 d of age in both male and female birds (Table 5, *P* > 0.05). The levels of Lys did not affect BWG, FI, and FCR except for FI in male chicks (*P* > 0.05). The birds fed 1.5% Lys had significantly lower FI in comparison with those fed 1.3% Lys.

*Experiment  2-2*. The effect of the level of CP was not significant on the performance of male or female birds (Table 6, *P* > 0.05); however, 23.2% CP improved BWG and FCR when compared with 21%. The level of Met + Cys did not affect neither male nor female birds' performance (*P* > 0.05), although the higher Met + Cys caused an improvement in BWG and FCR in both of sexes. In the male birds, there were significant differences among treatments in BWG and FI (*P* < 0.05), so that the chicks which received diet including 23.2% CP and 0.9% Met + Cys had higher BWG and FI.

## 4. Discussion

The results showed that the level CP had significant effect on BWG in 1–7 and 1–10 d of age (*P* < 0.05). With increasing CP level of diet, BWG increased. The result agreed with several investigators who showed that increased dietary protein content resulted in improved growth performance [19–24]. 

According to Darmani Kuhi et al. [25], the response of an animal to a limiting nutrient, generally follows the law of diminishing returns. This means that animal performance improvement improves in a nonlinear way with increasing dietary supplementation of the nutrient until the maximum potential growth of the animal, under the management conditions to which it is subjected, fully expressed and the greatest addition of the nutrient does not promote any additional response performance. The data obtained from the experiment revealed that chicks fed the diet containing 1.5% Lys recorded the highest values for FI and BWG. These findings were in accordance with those of Holsheimer and Ruesink [26], Kidd et al. [11], Aburto et al. [27], Kerr et al. [12] and Mukhtar et al. [28], who reported that increasing the dietary level of lysine or its consumption caused a progressive and significant increase (*P* < 0.05) in weight gain and the daily feed intake. These findings indicated that the lysine requirement as estimated by NRC [9] for 0 to 3 weeks old broilers, which is equal to 1.1%, may be low and for achieving better growth performance, increasing the dietary Lys level is needed.

The result of this study was in contrast with some previous studies. Cengiz et al. [29] reported that excess level of Lys in diet singly or in combination with excess level of Met, resulted in significant depression in BWG. They reported that Lys has been among the more toxic of the amino acids. Carew et al. [30] fed excess level of Lys (2.84 × above the NRC requirement) to chicks and found significant differences in performance parameters. These authors pointed out that excess level of Lys in broiler diets causes severe reduction in BWG (−25%) compared to the control group. In another study, Rezaei et al. [31] fed male broilers with graded levels of Lys (0, 1.5, 3 g/kg) from 0 to 6 weeks of age. In their study, BWG did not change significantly due to Lys addition at 0–3 weeks of age. Indeed they reported higher BWG by increasing Lys level in grower and total period of the experiment. The reason of growth performance differences among these data and those reported before might be attributable to factors such as Lys levels, time period of experiment, and consideration of Arg : Lys ratios in the diets.

Despite of interaction of (protein × Met + Cys) on FCR in 1–7 d and 1–10 d in experiment 1-2, there was no significant difference between FCR in treatment 3 (0.95% Met + Cys) and treatment 4 (1% Met + Cys).

In this study, in regard to positive interaction between protein and Met + Cys (in experiment 2-2), the treatments with higher level of CP (23.3%) and the lower level of Met + Cys (0.9%) showed better result of BWG and FI. This means that the level of 0.9% Met + Cys was adequate for those treatments. 

The results demonstrated that increasing TSAA from 0.85% to 1% had no significant effect on BWG, FI, and FCR during 1–7 and 1–10 d of age. Brito et al. [32], using diets with 20% CP and 0.641 and 0.926% Met + Cys, or 22% CP and 0.705 and 0.926% Met + Cys (total), found no differences between weight gain of broiler chicks during 1–7 day of age which is in agreement with the present study. Moreover, Sklan and Noy [15] observed maximum BW and feed efficiency to seven days of age chicks fed diets containing 0.91% Met + Cys (total). Leandro et al. [33], assessing the levels of Met (0.458, 0.507, 0.559, and 0.611%) at levels of 0.795, 0.847, 0.900, and 0.952% Met + Cys (total) in pre-starter diets for broiler chicks, found no significant response for FI, WG, and FCR, but the best results for weight at 7 days of age were tested at 0.900 and 0.952% Met + Cys.

The TSAA requirement estimates for chick performance in starter period in this study are in good agreement with the 1994 NRC estimated Met + Cys recommendation. The TSAA requirement as estimated by NRC [9] for 0 to 3 weeks old broilers is equal to 0.9%. These results are in agreement with those of Albino et al. [34], Raju et al. [35], De Oliveira Neto et al. [36], who reported that the levels of TSAA recommended by NRC [9] for starter diets of broiler chicks are sufficient.

## 5. Conclusion

A high level of CP in diet has positive effect on performance of broilers. The results indicate that the optimal added synthetic lysine level was 1.5%, but the level of 1.3% could be acceptable, too. It means that requirement of Lys to support obtaining the optimum of body weight and feed conversion ratio at early ages appear to be higher than suggested level by NRC [9]. 

Diet with 1% Met + Cys results in optimal performance, but the level of 0.9% could also be suggested which is in accordance with the NRC [9] recommendations for TSAA for starter diets.

## Figures and Tables

**Table 1:** The composition and calculated chemical analysis of diets in Experiment 1-1.

Ingredients (g/Kg)	Treatments							
	T1	T2	T3	T4	T5	T6	T7	T8
Corn	520	522	524	531	565	567	568	571
Soybean meal (44%)	376	375	374	368	338	337	336	335
Soy oil	35	34	33	32	28	27	27	25
Monocalcium phosphate	14	14	14	14	14	14	14	14
Limestone	16	16	16	16	16	16	16	16
Premix vit + min.^1, 2^	30	30	30	30	30	30	30	30
Salt	4	4	4	4	4	4	4	4

Calculated nutrients values								

Metabolizable energy (Kcal/Kg)	3000	3000	3000	3000	3000	3000	3000	3000
Crude protein (%)	23.2	23.2	23.2	23.2	21	21	21	21
Calcium (%)	1	1	1	1	1	1	1	1
Available phosphorus (%)	0.45	0.45	0.45	0.45	0.45	0.45	0.45	0.45
Na (%)	0.18	0.18	0.18	0.18	0.18	0.18	0.18	0.18
Arginine	1.39	1.39	1.39	1.38	1.30	1.29	1.29	1.29
Lysine (%)	1.5	1.4	1.3	1.2	1.5	1.4	1.3	1.2
Methionine + cystine (%)	1.0	1.0	1.0	1.0	1.0	1.0	1.0	1.0

^1^Each kg of premix provided vitamin A, 10000 IU; vitamin D3, 2500 IU; vitamin K, 2.4 mg; vitamin E, 44 IU; biotin, 0.1 mg; folic acid, 2.0 mg; niacin, 25 mg; calcium pantothenate, 14.32 mg; pyridoxine, 3.10 mg; ribo*ﬂ*avin, 5 mg; thiamin, 1.2 mg; vitamin B12, 10.5 *μ*g; Fe, 85 mg; Mn, 125 mg; Cu, 7.8 mg; Se, 0.09 mg; Zn, 60 mg; choline chloride, 5.5 mg.

^2^Lysine and methionine were added inside of premix.

**Table 2:** The composition and calculated chemical analysis of diets in Experiment 1-2.

Ingredients (g/Kg)	Treatments							
	T1	T2	T3	T4	T5	T6	T7	T8
Corn	522	521	521	520	566	565	565	565
Soybean meal (44%)	374	375	375	376	337	338	338	338
Soy oil	35	35	35	35	28	28	28	28
Monocalcium phosphate	16	16	16	16	16	16	16	16
Limestone	14.4	14.4	14.4	14.4	14.4	14.4	14.4	14.4
Premix vit + min.^1, 2^	30	30	30	30	30	30	30	30
Salt	4.6	4.6	4.6	4.6	4.6	4.6	4.6	4.6

Calculated chemical analysis								

Metabolizable energy (Kcal/Kg)	3000	3000	3000	3000	3000	3000	3000	3000
Crude protein (%)	23.2	23.2	23.2	23.2	21	21	21	21
Calcium (%)	1	1	1	1	1	1	1	1
Available phosphorus (%)	0.45	0.45	0.45	0.45	0.45	0.45	0.45	0.45
Na (%)	0.18	0.18	0.18	0.18	0.18	0.18	0.18	0.18
Arginine	1.39	1.39	1.39	1.39	1.39	1.39	1.39	1.39
Lysine (%)	1.5	1.5	1.5	1.5	1.5	1.5	1.5	1.5
Methionine + cystine (%)	0.85	0.9	0.95	1	0.85	0.9	0.95	1

^1^Each kg of premix provided vitamin A, 10000 IU; vitamin D3, 2500 IU; vitamin K, 2.4 mg; vitamin E, 44 IU; biotin, 0.1 mg; folic acid, 2.0 mg; niacin, 25 mg; calcium pantothenate, 14.32 mg; pyridoxine, 3.10 mg; ribo*ﬂ*avin, 5 mg; thiamin, 1.2 mg; vitamin B12, 10.5 *μ*g; Fe, 85 mg; Mn, 125 mg; Cu, 7.8 mg; Se, 0.09 mg; Zn, 60 mg; choline chloride, 5.5 mg.

^2^Lysine and methionine were added inside of premix.

**Table 3:** Effect of feeding diets containing various levels of crude protein (CP) and lysine (Lys) on body weight gain, feed intake, and feed conversion ratio of broiler chicks (Experiment 1-1).

Treatments			BWG (gr)		FI (gr)		FCR (gr/gr)	
Number	CP (%)	Lys (%)	1–7 d	1–10 d	1–7 d	1–10 d	1–7 d	1–10 d
1	23.2	1.5	223.7^a^	351.1^a^	190.3^a^	349.9	0.851	0.997
2	23.2	1.4	215.7^ab^	338.9^abc^	185.9^ab^	343.8	0.862	1.014
3	23.2	1.3	215.6^ab^	341.5^ab^	190.7^a^	350.1	0.885	1.025
4	23.2	1.2	205.7^bc^	324.9^cd^	180.0^b^	340.4	0.875	1.032
5	21	1.5	210.7^bc^	329.4^bcd^	187.2^ab^	344.2	0.888	1.045
6	21	1.4	209.1^bc^	329.6^bcd^	183.7^ab^	343.0	0.879	1.041
7	21	1.3	207.6^bc^	327.0^bcd^	184.9^ab^	339.7	0.891	1.039
8	21	1.2	204.1^c^	316.4^d^	178.9^b^	333.2	0.877	1.053
Pooled SEM			3.05	4.41	2.39	4.21	0.016	0.015

Probability								

Protein × lysine			*	*	*	NS	NS	NS
Protein			*	*	NS	NS	NS	*
Lysine			*	*	*	NS	NS	NS

^a–d^Means in each column without common superscripts are significantly different.

*Data are means of four replicates having 20 birds in each. BWG: body weight gain, FI: feed intake, FCR: feed conversion ratio, CP: crude protein, Lys: lysine, SEM: standard error of mean, NS: not statistically significant; *P < 0.05.

**Table 4:** Effect of feeding diets containing various levels of crude protein (CP) and methionine + cystine (Met + Cys) on body weight gain, feed intake, and feed conversion ratio of broiler chicks (Experiment 1-2).

Treatments			BWG (gr)		FI (gr)		FCR (gr/gr)	
Number	CP (%)	Met + Cys (%)	1–7 d	1–10 d	1–7 d	1–10 d	1–7 d	1–10 d
1	23.2	0.85	214.5	332.2^a^	188.8	351.3^a^	0.880^a^	1.057^ab^
2	23.2	0.90	217.8	341.8^a^	184.4	352.1^a^	0.847^ab^	1.030^bc^
3	23.2	0.95	215.4	340.4^a^	177.2	333.0^b^	0.823^bc^	0.978^c^
4	23.2	1.00	222.0	346.7^a^	178.5	337.5^b^	0.804^c^	0.973^c^
5	21	0.85	216.3	337.4^a^	182.6	343.4^ab^	0.844^ab^	1.018^bc^
6	21	0.90	213.3	330.8^a^	185.0	345.0^ab^	0.867^a^	1.043^ab^
7	21	0.95	208.8	312.5^b^	180.6	337.0^b^	0.865^a^	1.078^a^
8	21	1.00	214.0	333.6^a^	183.5	340.6^ab^	0.857^ab^	1.021^bc^
Pooled SEM			2.45	4.43	2.89	4.71	0.011	0.011

Probability								

Protein × (Met + Cys)			NS	*	NS	*	**	*
Protein			NS	NS	NS	NS	NS	NS
Met + Cys			NS	NS	NS	NS	NS	NS

^a–d^Means in each column without common superscripts are significantly different.

*Data are means of four replicates having 20 birds in each replicate. BWG: body weight gain, FI: feed intake, FCR: feed conversion ratio, CP: crude protein, Met + Cys: methionine + cystine, SEM: standard error of mean, NS: not statistically significant; *P < 0.05; **P < 0.01.

**Table 5:** Effect of feeding diets containing two levels of crude protein (CP) and lysine (Lys) on body weight gain, feed intake and feed conversion ratio of male or female broiler chicks at 10 d (Experiment 2-1).

Treatments			BWG (g)		Feed intake (g)		FCR (g/g)	
No.	CP (%)	Lys (%)	male	female	male	female	male	female
1	23.2	1.5	336.8	327.4	339.3	335.9	1.007	1.026
2	23.2	1.3	327.5	321.4	344.0	330.5	1.051	1.028
3	21	1.5	322.2	320.4	337.8	335.2	1.049	1.046
4	21	1.3	323.1	309.5	342.3	326.3	1.059	1.054
Pooled SEM			4.22	4.05	2.97	4.50	0.010	0.015

Probability								

Protein × Lysine			NS	NS	NS	NS	NS	NS
Protein			NS	NS	NS	NS	NS	NS
Lysine			NS	NS	*	NS	NS	NS

^a–d^Means in each column without common superscripts are significantly different.

*Mean of four replicates having 50 birds. BWG: Body weight gain, FI: Feed intake, FCR: Feed conversion ratio, CP: Crude protein, Lys: Lysine; SEM Standard error of mean; NS: Not-statistically significant; *P < 0.05.

**Table 6:** Effect of feeding diets containing two levels of crude protein (CP) and methionine + cystine (Met + Cys) on body weight gain, feed intake and feed conversion ratio of male or female broiler chicks at 10 d (Experiment 2-2).

Treatments			BWG (g)		Feed intake (g)		FCR (g/g)	
No.	CP (%)	Met + Cys (%)	male	female	male	female	male	female
1	23.2	1.00	323.2^ab^	322.3	334.8^b^	332.6	1.037	1.032
2	23.2	0.90	330.5^a^	318.3	344.7^a^	336.4	1.043	1.057
3	21	1.00	326.5^a^	317.4	340.0^ab^	330.1	1.042	1.041
4	21	0.90	312.5^b^	318.1	339.5^ab^	330.6	1.087	1.039
Pooled SEM			3.1	3.87	2.3	3.37	0.017	0.012

Probability								

Protein × (Met + Cys)			*	NS	*	NS	NS	NS
Protein			NS	NS	NS	NS	NS	NS
Met + Cys			NS	NS	NS	NS	NS	NS

^a–d^Means in each column without common superscripts are significantly different.

*Data are means off our 4 replicates having 50 birds. BWG: Body weight gain, FI: Feed intake, FCR: Feed conversion ratio, CP: Crude protein, Met + Cys: Methionine + Cystine, NS: Not-statistically significant; *P < 0.05.
